# Tuning Texture and Morphology of Mesoporous TiO_2_ by Non-Hydrolytic Sol-Gel Syntheses

**DOI:** 10.3390/molecules23113006

**Published:** 2018-11-17

**Authors:** Yanhui Wang, Maroua Bouchneb, Johan G. Alauzun, P. Hubert Mutin

**Affiliations:** Institut Charles Gerhardt, CNRS-UM-ENSCM, Université Montpellier, 34095 Montpellier, France; yanhui.wang@umontpellier.fr (Y.W.); maroua.bouchneb@etu.umontpellier.fr (M.B.); johan.alauzun@univ-montp2.fr (J.G.A.)

**Keywords:** non-hydrolytic sol-gel, TiO_2_, mesoporosity

## Abstract

The development of powerful synthetic methodologies is paramount in the design of advanced nanostructured materials. Owing to its remarkable properties and low cost, nanostructured TiO_2_ is widely investigated for applications such as photocatalysis, energy conversion or energy storage. In this article we report the synthesis of mesoporous TiO_2_ by three different non-hydrolytic sol-gel routes, and we investigate the influence of the synthetic route and of the presence and nature of the solvent on the structure, texture and morphology of the materials. The first route is the well-known ether route, based on the reaction of TiCl_4_ with ^i^Pr_2_O. The second and third routes, which have not been previously described for the synthesis of mesoporous TiO_2_, involve the reaction of Ti(O^i^Pr)_4_ with stoichiometric amounts of acetophenone and benzoic anhydride, respectively. All materials are characterized by XRD, N_2_ physisorption and SEM. By playing with the non-hydrolytic route used and the reaction conditions (presence of a solvent, nature of the solvent, calcination), it is possible to tune the morphology and texture of the TiO_2_. Depending on the reaction conditions, a large variety of mesoporous TiO_2_ nanostructures could be obtained, resulting from the spontaneous aggregation of TiO_2_ nanoparticles, either rounded nanoparticles, platelets or nanorods. These nanoparticle networks exhibited a specific surface area up to 250 m^2^ g^−1^ before calcination, or up to 110 m^2^ g^−1^ after calcination.

## 1. Introduction

Owing to its outstanding properties, low toxicity and low cost, TiO_2_ has been attracting tremendous attention for environmental and energy applications [1] such as photocatalysis [2,3,4], catalysis [5], water-splitting [6], sensing [7] and energy conversion and storage [8,9,10,11].

The development of powerful synthetic methods plays a pivotal role in the design of advanced materials and much research has been dedicated to the synthesis of TiO_2_ nanomaterials. The sol-gel process is the most common method used to synthesize metal oxides. The conventional methods are based on hydrolysis and condensation but, following the pioneering work of Vioux’s group [12,13], several alternative non-hydrolytic (or non-aqueous) sol-gel routes have been developed to avoid the disadvantages of hydrolytic sol-gel, such as high hydrolysis-condensation rates of metal alkoxides, low degree of condensation, and formation of poorly crystalline oxo-hydroxides [14,15,16,17,18]. Non-hydrolytic sol-gel (NHSG) is performed in an organic medium and involves organic oxygen-donors, such as ethers or alcohols, instead of water. NHSG has been established as a powerful methodology for the synthesis of mesoporous oxide or mixed oxide xerogels [19,20] and of crystalline metal oxide nanoparticles [18].

Several NHSG routes have been used to prepare TiO_2_ nanomaterials, notably the alkoxide route and ether routes based on the reaction at 40–150 °C of a titanium halide precursor (typically TiCl_4_) with a stoichiometric amount of a titanium alkoxide (usually Ti(O^i^Pr)_4_ or an ether (usually diisopropylether) [13,21]. These so-called alkoxide and ether routes have been successfully used to prepare mesoporous TiO_2_-based materials [22,23,24] and nanoparticles [25,26,27,28].

Alcohols (e.g., isopropanol, tert-butanol or benzyl alcohol) have also been used as oxygen donors [21,29,30]. In particular, the benzyl alcohol route, based on the reaction of various Ti precursors (TiCl_4_, Ti(O^i^Pr)_4_) with excess of benzyl alcohol acting as solvent, oxygen donor and surface ligand, has been extensively used to prepare TiO_2_ nanoparticles in the absence of a capping agent [30,31,32].

The NHSG synthesis of TiO_2_ by the reaction of Ti(O^i^Pr)_4_ with acetic anhydride was also attempted, but this reaction was found to be quite slow, even at 140 °C, and catalysis by TiCl_4_ was required [21]. Anatase nanoparticles were obtained by the reaction of Ti(O^i^Pr)_4_ with various ketones (e.g., acetone, acetophenone) or aldehydes (e.g., butyraldehyde, benzaldehyde) [33,34].

The objective of this work is to compare the synthesis of mesoporous TiO_2_ by three different NHSG routes, and we explore the influence of the synthetic route and of the presence and nature of the solvent on the structure, texture and morphology of the resulting nanomaterials. The first route is the ether route, based on the reaction of TiCl_4_ with ^i^Pr_2_O; this route has been extensively used for the synthesis of TiO_2_, but the synthesis in non-polar solvents such as toluene, squalane or cyclohexane has not been reported. The second and third routes, which have not been previously described for the synthesis of mesoporous TiO_2_, involve the reaction of Ti(O^i^Pr)_4_ with stoichiometric amounts of acetophenone and benzoic anhydride, respectively.

## 2. Results

### 2.1. Synthesis of TiO_2_ Samples and Reactions Involved

The synthesis of the different TiO_2_ samples is detailed in the ‘Materials and Methods’ part. In all cases the precursor was reacted with 2 equivalents of the oxygen donor under autogenous pressure in a Teflon lined stainless steel autoclave. The samples were labeled TiO_2_-X-Y, where X corresponds to the oxygen donor (E, A and B stand for diisopropylether, acetophenone and benzoic anhydride, respectively) and Y corresponds to the solvent (NS, Tol, Squ and CH stand for no solvent, toluene, squalane, and cyclohexane, respectively), as shown in Table 1. The yields upon calcination are very high (>90%) for the samples prepared by the ether and acetophenone routes, indicating very high degrees of condensation. Conversely, the calcination yields are much lower for the samples prepared by the benzoic anhydride route (53–78%), pointing to significantly lower degrees of condensation. 

**Scheme 1:**
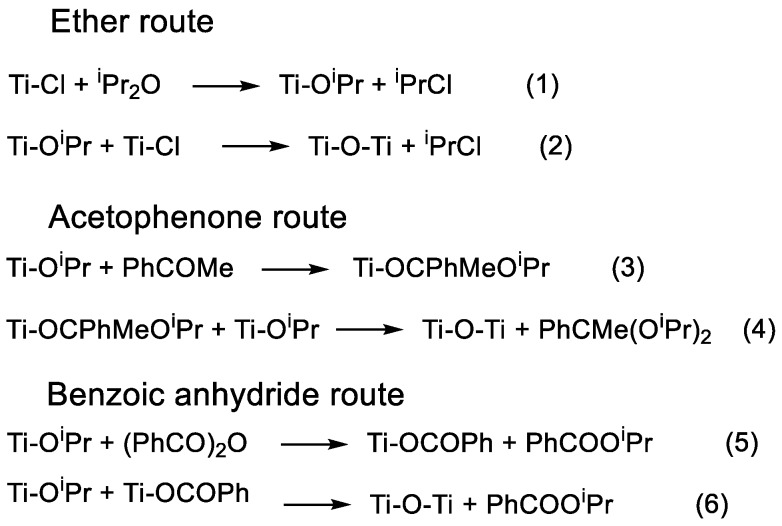
Proposed reaction schemes for the NHSG routes used in this work.

The reactions likely involved in the NHSG routes used in this work are summarized in Scheme 1. The ether route based on the reaction of diisopropylether (^i^Pr_2_O) and TiCl_4_ is well documented; it involves an etherolysis (Equation (1)) and a condensation step (Equation (2)), with, in both cases, elimination of isopropyl chloride [21]. The acetophenone route has been described previously for the synthesis of TiO_2_ [33] and BaTiO_3_ [35] nanoparticles, using acetophenone as a solvent and an O-donor. However, in the present case, a stoichiometric amount of acetophenone was used (2 equivalents relative to Ti(O^i^Pr)_4_). According to Pazik et al., the main pathway could involve the elimination of the ketal compound PhCMe(O^i^Pr)_2_ (Equations (3) and (4)). Benzoic anhydride has not been used previously to prepare metal oxides, but the mechanism should be similar to the one reported for acetic anhydride, with formation of the ester PhCOO^i^Pr (Equations (5) and (6)) [21].

### 2.2. Structural and Textural Characterization of TiO_2_ Samples

#### 2.2.1. Powder X-Ray Diffraction (XRD)

**Figure 1:**
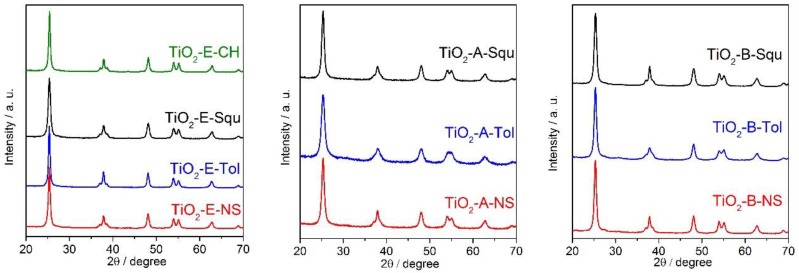

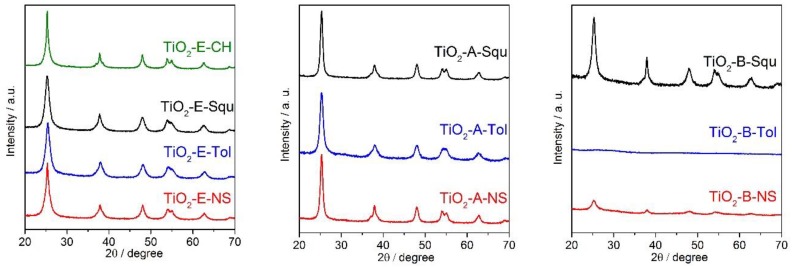
Powder X-ray diffraction (XRD) patterns of non-calcined (**bottom**) and calcined (**top**) TiO_2_ samples.

The powder XRD patterns of the TiO_2_ samples before and after calcination (5 h at 500 °C in air) are displayed in Figure 1. The only crystalline phase detected was the anatase phase (JCPDS 21-1272). All samples prepared by the ether or the acetophenone routes appeared well crystallized, even before calcination. Conversely, the samples prepared by the benzoic anhydride route were poorly crystallized before calcination (TiO_2_-B-Squ or TiO_2_-B-NS) or even amorphous to XRD (TiO_2_-B-Tol). 

The crystallite size, evaluated by the Scherrer equation for the (101) reflection, ranged from 7 to 13 nm for the non-calcined samples and from 9 to 28 nm after calcination (Table 2). The grain growth upon calcination was significant for the samples prepared by the ether route (e.g., from 9 to 23 nm for TiO_2_-E-NS), while it was limited for the samples prepared by the acetophenone route (e.g., from 11 to 12 nm for TiO_2_-A-NS).

#### 2.2.2. Nitrogen Physisorption

**Figure 2:**
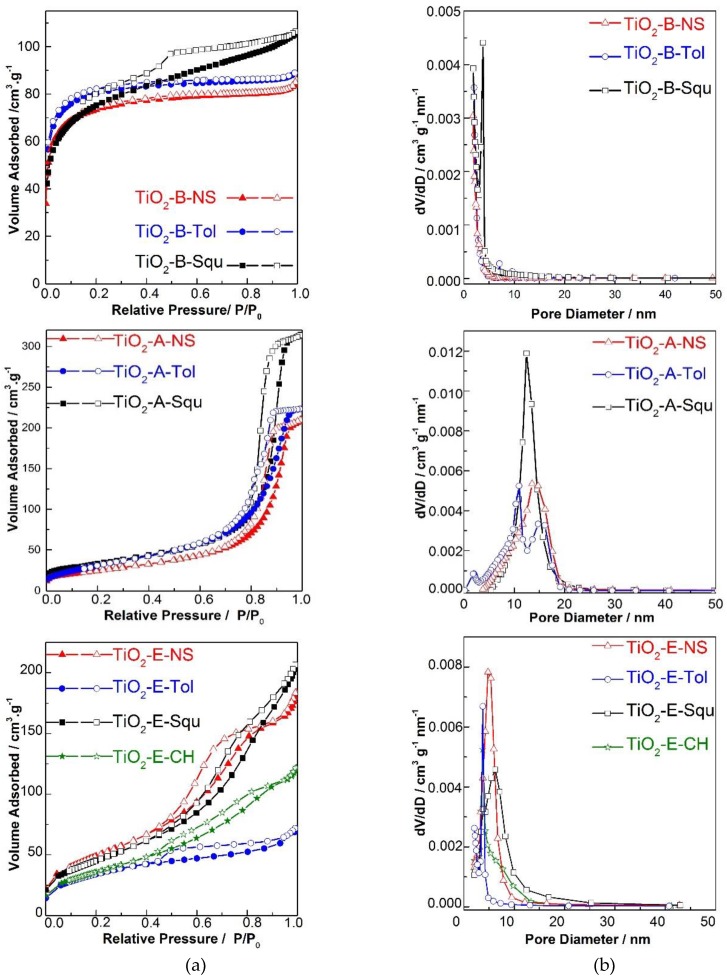
Nitrogen physisorption isotherms (**a**) and pore size distribution (**b**) of non-calcined TiO_2_ samples. Filled and open symbols refer to adsorption and desorption, respectively.

**Figure 3:**
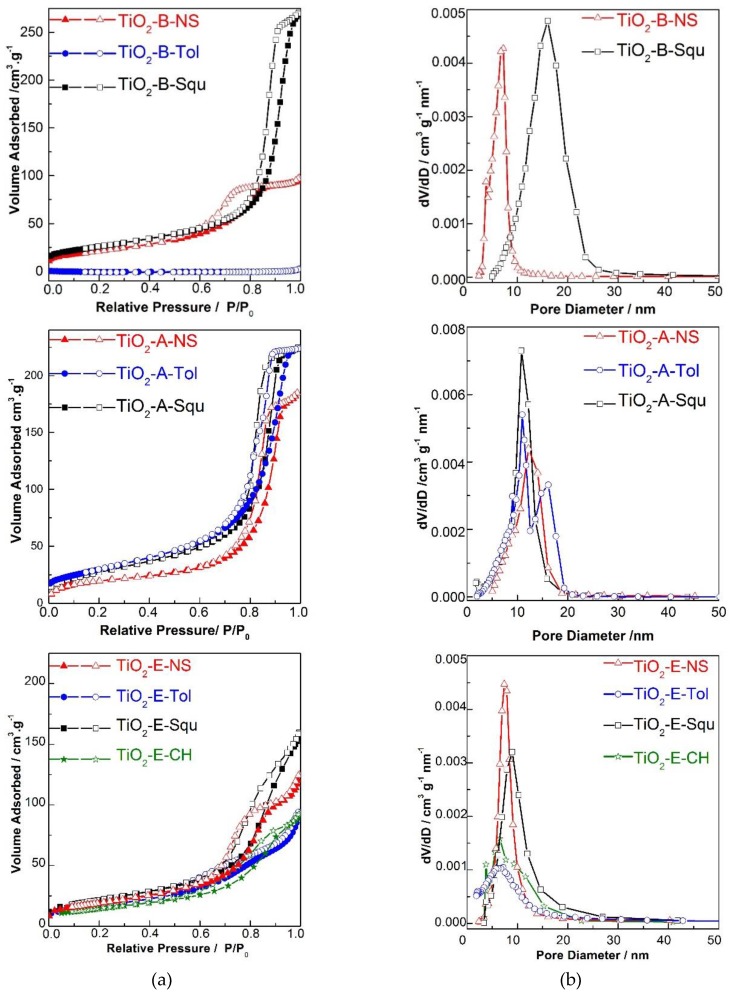
Nitrogen physisorption isotherms (**a**) and pore size distribution (**b**) of calcined TiO_2_ samples. Filled and open symbols refer to adsorption and desorption, respectively.

The texture of the TiO_2_ samples before and after calcination was investigated using nitrogen physisorption at 77 K. Typical textural data (BET specific surface area, total pore volume and BJH average pore diameter) are displayed in Table 2. The adsorption−desorption isotherms and pore size distributions of the non-calcined and calcined samples are given in Figure 2 and Figure 3, respectively.

Except for the calcined TiO_2_-B-Tol sample, which is non porous, all the samples show significant specific surface areas ranging from 90 to 250 m^2^ g^−1^ for the non-calcined samples and from 50 to 110 m^2^ g^−1^ for the calcined ones. 

According to the IUPAC classification [36], the isotherms of the calcined porous samples are either of type IVa (well-defined mesoporosity, e.g., calcined TiO_2_-A-NS, TiO_2_-A-Tol, TiO_2_-A-Squ, TiO_2_-B-NS) or are composite Type IVa + Type II isotherms (e.g., calcined TiO_2_-E-NS, TiO_2_-E-Tol, TiO_2_-E-squ, TiO_2_-E-CH), indicating the presence of both well-defined mesopores and macropores [36]. 

BJH analysis confirms the presence of mesopores with average diameters ranging from ca 3 to 30 nm, depending on the NHSG route used and on the solvent. The isotherms of the non-calcined samples (Figure 2) are similar to those of the calcined samples, with the exception of the samples prepared by the benzoic anhydride route which show type I or type I + IV isotherms, indicating the presence of micropores.

### 2.3. Morphology of TiO_2_ Samples

**Figure 4:**
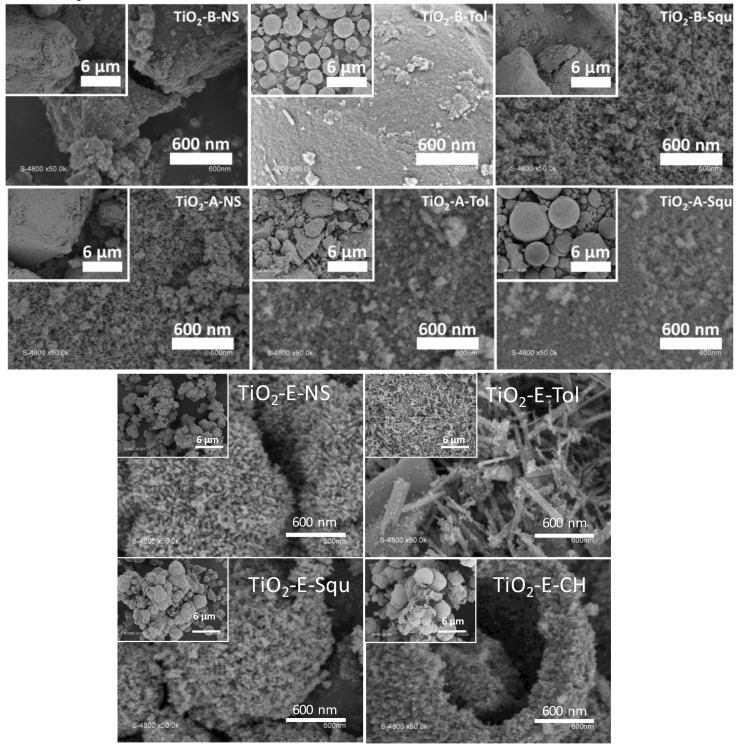
SEM images of calcined TiO_2_ samples.

As shown in Figure 4, the morphology of the calcined samples depends both on the oxygen donors and the solvents used. The primary nanoparticles exhibit different morphologies and size. In most cases they are well-calibrated, roughly spherical nanoparticles with sizes ranging from ≈5 to 15 nm, but can be also platelets ≈50 nm in width and ≈10 nm thick (TiO_2_-E-NS) or rods up to 1 µm in length (TiO_2_-E-Tol). These primary nanoparticles either form large, more or less compact aggregates (e.g., TiO_2_-E-Tol, TiO_2_-A-NS, TiO_2_-A-Tol, TiO_2_-B-NS, TiO_2_-B-Squ) or form spherical secondary aggregates, ≈1 to 15 µm in size (e.g., TiO_2_-E-NS, TiO_2_-E-Squ, TiO_2_-E-CH, TiO_2_-A-Squ, TiO_2_-B-Tol).

In the case of the TiO_2_-B-Tol sample, prior to calcination (Figure S1), the particles were very small but not sintered together, whereas after calcination (Figure 4) the sintered nanoparticles formed highly compact secondary aggregates, accounting for the negligible specific surface area and porosity of this sample.

## 3. Discussion

The aim of this article was to investigate the influence of different NHSG routes and of the presence and nature of the solvent on the structure, texture and morphology of the materials. The first route is the well-known ether route, based on the reaction of TiCl_4_ with ^i^Pr_2_O. The second and third routes, which have not been previously described for the synthesis of mesoporous TiO_2_, involve the reaction of Ti(O^i^Pr)_4_ with stoichiometric amounts of acetophenone and benzoic anhydride, respectively. 

One important outcome of this work is that the acetophenone and benzoic anhydride routes offer efficient ways to obtain mesoporous TiO_2_ materials, starting from the commercially available, easy to handle, Ti(O^i^Pr)_4_ precursor. For applications such as catalysis or sensing, the presence of residual chloride groups may be a major drawback, thus avoiding the use of TiCl_4_ as a precursor can be important. Additionally, the HCl released by the hydrolysis of chloride groups is highly corrosive.

In all three routes a stoichiometric amount of O-donor is used and solvent is not necessary, which from the point of view of atom economy is interesting. Nevertheless, playing with the route and reaction conditions offers simple and reproducible ways to tune the morphology and texture of the final TiO_2_. 

**Scheme 2:**
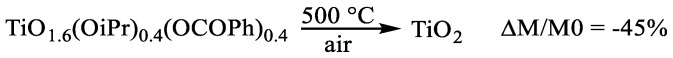
Theoretical weight loss for a TiO_2_ xerogel with a degree of condensation of ≈60% (assuming an equal number of O^i^Pr and O(CO)Ph residual groups and complete conversion to TiO_2_).

The calcination yields (Table 1) indicate very high degrees of condensation in the ether and acetophenone routes, and lower ones in the benzoic anhydride route. For instance, the ≈55% calcination yield of TiO_2_-B-NS and TiO_2_-B-Tol would correspond to a degree of condensation of ≈60% assuming an equal number of O^i^Pr and O(CO)Ph residual groups and complete conversion to TiO_2_, as shown in Scheme 2.

Whatever the route and the solvent used in the synthesis, the only crystalline phase found in the resulting TiO_2_ samples is the anatase phase, which is the most commonly obtained TiO_2_ polymorph in non-hydrolytic sol-gel synthesis (although formation of rutile and brookite has been reported in the reaction of TiCl_4_ with some alcohols) [21]. The samples prepared by the benzoic anhydride route were poorly crystallized before calcination, particularly the TiO_2_-B-Tol and TiO_2_-B-NS samples. This behavior is quite uncommon, as NHSG syntheses are noted for yielding well-crystallized metal oxides even before calcination, and is likely related to the low degree of condensation of these xerogels.

The morphology of the calcined samples is very much dependent on the reaction conditions. The shape and size of the primary particles is more varied in the ether route (spheres, platelets or nanorods), whereas only spherical primary particles are observed in the other routes. Formation of micron-sized, spherical secondary particles is observed in all the routes (TiO_2_-B-Tol, TiO_2_-A-Squ, TiO_2_-B-NS, TiO_2_-E-Squ and TiO_2_-E-CH), but there is no correlation with the nature of the solvent. Actually, it is well known in nanoparticle synthesis that both the morphology and arrangement of the crystallites can be tuned by the addition of small amounts of ligands. In our case, the different morphologies observed are likely governed by the nature of the residual groups and reaction by-products, which depends on the route, and also by the properties of the solvent. As previously noted for the non-hydrolytic benzyl alcohol route [15], this sensitivity to the reaction conditions offers the possibility to tune the morphology of the samples, but predicting the morphology is not yet possible.

The same applies to the texture of the TiO_2_ samples: depending on the route used and on the reaction conditions, materials with a wide range of specific surface areas and pore sizes can be obtained.

The specific surface area of a network built of non-porous nanoparticles depends on the size and shape of the primary nanoparticles and on their degree of sintering. As a result, the texture is sensitive to the calcination treatment, which may lead to particle growth and sintering. In the case of samples prepared by the diisopropylether or benzoic anhydride routes (except TiO_2_-B-Tol), calcination leads to a decrease of the specific surface area by a factor of 2 to 3; this decrease can be mostly ascribed to the significant increase in crystallite size taking place during the calcination. Similarly, the loss of specific surface area upon calcination is moderate (decrease by a factor of 1.2 to 1.4) for the samples prepared by the acetophenone route which show very limited grain growth. The geometric specific surface area of monodisperse spherical anatase particles (non sintered) is given by the relationship S_geom_ = 6000/(ρ.D), where ρ is the specific gravity (in g cm^−3^) and D the diameter (in nm). For example, for anatase (ρ = 3.9 g cm^−3^), this relationship gives S_geom_ values ranging from 154 to 62 m^2^ g^−1^ for particle diameters ranging from 10 to 25 nm. In most cases, the specific surface area of the calcined samples is 5 to 40% lower than the geometric surface area estimated from their crystallite size (from XRD data), indicating low to moderate sintering. The behavior of the sample TiO_2_-B-Tol is outstanding. Prior to calcination, this sample was amorphous and exhibited a very high specific surface area (250 m^2^ g^−1^); after calcination, the specific surface area decreased to less than 10 m^2^ g^−1^, although the anatase nanocrystals formed were quite small (13 nm). This indicates complete sintering of the particles growing from an amorphous phase.

## 4. Materials and Methods 

All manipulations were carried out under an argon atmosphere in a glovebox (<10 ppm H_2_O and O_2_). Titanium (IV) chloride (TiCl_4_ 99%), titanium (IV) isopropoxide (Ti(O^i^Pr)_4_ 97%), acetic anhydride (Ac_2_O, 99%) and acetophenone (99%) were obtained from Sigma-Aldrich (St.Quentin Fallavier, France). Squalane (98%) was purchased from Alfa Aesar (Karlsruhe, Germany). Diisopropylether (^i^Pr_2_O) and toluene (Sigma-Aldrich 99.7%) were dried over a Pure Solve MD5 solvent purification system (H_2_O < 10 ppm). Cyclohexane (Sigma-Aldrich 99.5%) was dried over molecular sieves. All other chemicals were used without further purification.

TiO_2_ samples were prepared without solvent or in the presence of a solvent (toluene, squalane or cyclohexane). The precursor (TiCl_4_ or Ti(O^i^Pr)_4_), the oxygen donor (2 equivalents relative to the precursor) and the solvent (if any) were mixed in a stainless steel digestion vessel equipped with a PTFE lining (23 mL). Then the sealed autoclave was heated in an oven under autogenous pressure. The reaction conditions are summarized in Table 1. After reaction, the resulting white precipitate was thoroughly washed with CH_2_Cl_2_ (Carlo Erba 99.8%) (TiO_2_-E samples) or acetone (TiO_2_-A and TiO_2_-B samples). The precipitate was then dried under vacuum at room temperature for 5 h and then ground into a fine white powder. Calcination was carried out in a muffle furnace at 500 °C (heating rate 5 °C/min) for 5 h in ambient air. 

X-Ray diffraction (XRD) patterns were recorded with Cu_K*α*_ radiation (*λ* = 1.5418 Å) on a PANalytical X’Pert Pro MPD diffractometer (Royston, United Kingdom). Crystallite size was estimated from the most intense reflection (25.28° 2θ) using the Scherrer equation. Scanning electron microscopy images were obtained with a Hitachi S-4800 electron microscope. N_2_-physisorption experiments were performed at −196 °C on a Micromeritics TriStar 3000 (Merignac, France). The samples were de-gassed under vacuum at 180 °C for 15 h prior to measurement. The total pore volume was measured at *P*/*P*_0_ > 0.985. The pore size distribution was derived by the BJH method from the desorption branch.

## 5. Conclusions

A set of ten TiO_2_ samples was prepared by three different non-hydrolytic sol-gel routes, and the influence of the reaction conditions (presence of a solvent, nature of the solvent, calcination) on the structure, texture and morphology of the samples was investigated. The acetophenone route appears to be a promising, chloride-free, non-hydrolytic sol-gel route, giving well-crystallized anatase materials even before calcination and with very interesting mesoporous textures compared to the well-known ether route. The benzoic anhydride route appears less promising due to the low crystallinity and significant microporosity of the non-calcined samples and the dramatic loss of specific surface area upon calcination. In each route, playing with the presence and nature of the solvent offers the possibility to tune the morphology of the samples, but prediction is not yet possible.

## Figures and Tables

**Table 1:** Reaction conditions for the synthesis of the different TiO_2_ samples.

Sample	Precursor (amount)	O-donor ^a^	Solvent	Temperature (time)	Calc. yield ^b^/%
TiO_2_-E-NS	TiCl_4_ (12.5 mmol)	^i^Pr_2_O	none	110 °C (72 h)	93
TiO_2_-E-Tol	TiCl_4_ (12.5 mmol)	^i^Pr_2_O	toluene (5 mL)	110 °C (72 h)	94
TiO_2_-E-Squ	TiCl_4_ (12.5 mmol)	^i^Pr_2_O	squalane (5 mL)	110 °C (72 h)	96
TiO_2_-E-CH	TiCl_4_ (12.5 mmol)	^i^Pr_2_O	cyclohexane (5 mL)	110 °C (72 h)	93
TiO_2_-A-NS	Ti(O^i^Pr)_4_ (4.4 mmol)	PhCOCH_3_	none	200 °C (12 h)	92
TiO_2_-A-Tol	Ti(O^i^Pr)_4_ (4.4 mmol)	PhCOCH_3_	toluene (10 mL)	200 °C (12 h)	95
TiO_2_-A-Squ	Ti(O^i^Pr)_4_ (4.4 mmol)	PhCOCH_3_	squalane (10 mL)	200 °C (12 h)	96
TiO_2_-B-NS	Ti(O^i^Pr)_4_ (4.4 mmol)	(PhCO)_2_O	none	200 °C (12 h)	56
TiO_2_-B-Tol	Ti(O^i^Pr)_4_ (4.4 mmol)	(PhCO)_2_O	toluene (10 mL)	200 °C (12 h)	53
TiO_2_-B-Squ	Ti(O^i^Pr)_4_ (4.4 mmol)	(PhCO)_2_O	squalane (10 mL)	200 °C (12 h)	78

^a^ 2 equivalents relative to the precursor; ^b^ calcination yield (500 °C, 5 h, in air).

**Table 2:** Crystallite size and textural data for the different TiO_2_ samples, before and after calcination.

Sample	Calcination	Cryst. size ^a^ (nm)	S_BET_^b^ (m^2^ g^−1^)	Vp ^c^ (cm^3^ g^−1^)	Vmeso ^d^ (cm^3^ g^−1^)	Dp ^e^ (nm)
TiO_2_-E-NS	no yes	9 23	180 70	0.28 0.19	0.27 0.18	5 9
TiO_2_-E-Tol	no yes	8 28	130 60	0.10 0.15	0.08 0.15	4 7
TiO_2_-E-Squ	no yes	8 17	170 80	0.31 0.24	0.31 0.23	7 10
TiO_2_-E-CH	no yes	13 28	130 50	0.18 0.14	0.17 0.14	5 9
TiO_2_-A-NS	no yes	11 12	90 70	0.32 0.29	0.32 0.28	10 12
TiO_2_-A-Tol	no yes	9 9	120 110	0.35 0.35	0.35 0.35	10 11
TiO_2_-A-Squ	no yes	12 13	120 100	0.48 0.35	0.48 0.34	13 10
TiO_2_-B-NS	no yes	9 12	240 80	0.13 0.15	0.02 0.15	3 6
TiO_2_-B-Tol	no yes	am. 13	250 <10	0.14 <0.05	0.03 NA	3 NA
TiO_2_-B-Squ	no yes	7 14	240 90	0.16 0.42	0.07 0.42	4 15

^a^ crystallite size estimated by the Scherrer equation; ^b^ BET specific surface area; ^c^ total pore volume at *P*/*P*_0_ = 0.99; ^d^ BJH volume of pores in the 2 to 50 nm range, calculated from the desorption branch. ^e^ BJH average mesopore diameter calculated from the desorption branch.

## Supplementary Materials

The following are available online. Figure S1: SEM images of non-calcined TiO_2_ samples.
