# Supplementary figures and images for: Tuning Texture and Morphology of Mesoporous TiO2 by Non-Hydrolytic Sol-Gel Syntheses
Source: Molecules. 2018 Nov 17;23(11):3006. doi: 10.3390/molecules23113006 (PMC6278356; doi:10.3390/molecules23113006)

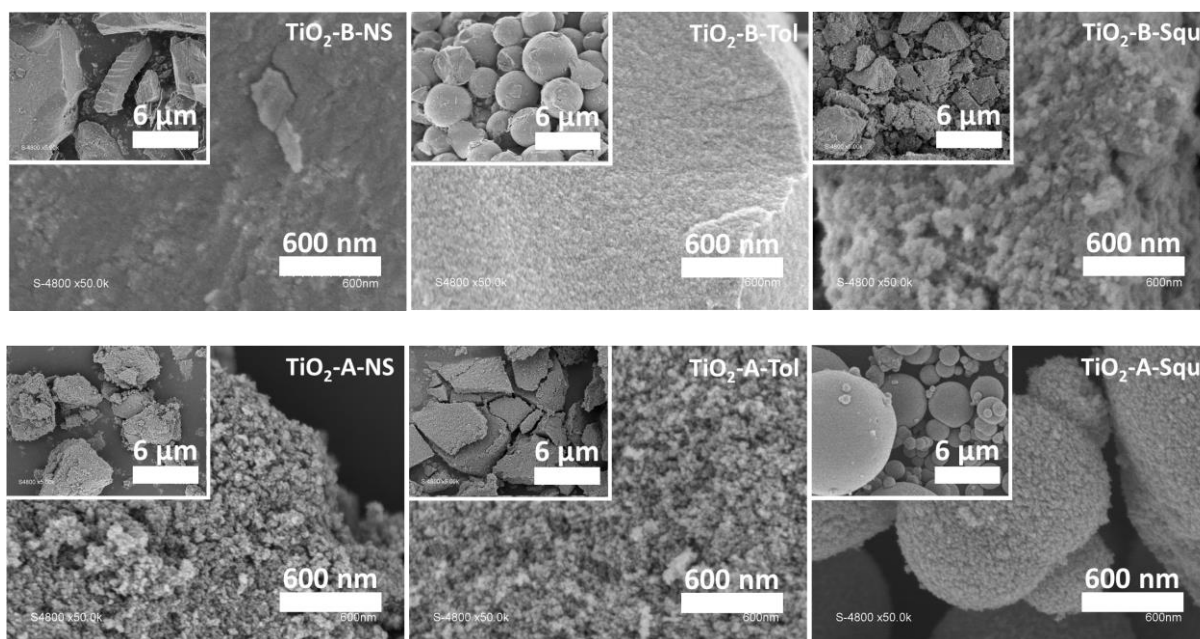

**Figure S1:** SEM images of non-calcined  $\text{TiO}_2$  samples

Supplement: Supplementary file 1 [file molecules-23-03006-s001.pdf]
